# Editorial: Global Health and Medical Travel

**DOI:** 10.3389/fpubh.2016.00235

**Published:** 2016-10-21

**Authors:** Kai Ruggeri, Saba Hinrichs-Krapels

**Affiliations:** ^1^Policy Research Group, Department of Psychology, University of Cambridge, Cambridge, UK; ^2^The Policy Institute, King’s College London, London, UK

**Keywords:** global health, medical travel, health-care access, health policy, health services

**The Editorial on the Research Topic**

**Global Health and Medical Travel**

Travel for medical care is increasing globally. This ranges from patients with urgent medical needs who must travel abroad for specialist care, to medical tourists who elect to have certain procedures abroad. While estimated increases of patients traveling abroad for medical care vary, more empirical research and evidence is needed to understand the scale, reasons, and consequences of travel. The preceding Research Topic has been compiled to reflect significant gaps in evidence and policy specifically for medical travel, referring here to care that is essential to maintain quality of life, largely influenced by the Global Health Access Policy (GHAP) project, which was carried out from 2012 to 2015.

The editors had previously called for more scientific evidence to support the development of such policies and more nuanced definitions rather than interchangeable use of “medical tourism” and “medical travel” (1). We noted that, prior to the GHAP project, research on medical travel had generally been limited to medical tourism or simplified exchanges between few locations over a limited scope of care. Studies lacked extensive review required to inform medical travel policies for those with urgent needs and did not consider factors such as patient protection and indemnification, psychological support during rehabilitation, and long-term liability (2). For this reason, this Topic was convened to invite studies that contribute evidence toward the development of such policies specific to medical travel, which emphasizes more critical care than much of the work on medical tourism. The following is a summary of arguments made therein.

## International Quality Standards

Myriad treatments may prompt medical travel, each of which have different pricing implications and address a variety of needs. Judgment on whether to include specific treatment policies requires direct quality assessment of those treatments. Existing international standards, such as the OECD Health Care Quality Indicators, are more conceptual and therefore lack the detail necessary to ensure specific procedural standards. The Perspective by Kácha et al. suggests the domains included in such a framework for international quality standards should include minimum quality standards globally, financial responsibility for all prior to and following care, patient centeredness, and consideration of local cultural challenges. They suggest prioritizing more specific standards by procedure and establishing comparable safety and quality indicators.

## Patient Choice and Medical Travel

When treatment is unavailable (e.g., not offered locally, excessive waiting list) or particularly difficult to access (e.g., high cost, poor quality), medical travel is an increasingly common option. Though studies have identified factors in decision-making processes regarding medical travel, it is not clear which have the greatest influence and how they may interact to influence outcomes. Such insights are necessary to understand choice. The article by Zhukovsky et al. surveying patient choice among a predominantly well-educated, European sample showed that individuals display high willingness to travel for medical care. They also found that cost was less important than quality and waiting time for treatment. As the authors note, if treated carefully, insights into influences on patient choice could be valuable in establishing relevant, population-appropriate policies. Further research must identify locations involved and how these affect reasons for traveling among larger samples, especially for future insight into specific treatments.

## Legal Frameworks

Increased medical travel may result in uncontrolled movement of patients. This carries significant risks for both patients and health-care systems with respect to quality standards, spread of disease, financial implications of adverse events, and unregulated health-care markets (3, 4). Globally, however, medical travel remains a widely unregulated extension of health care. In one Perspective, we seek to address existing gaps in the regulation of medical travel by identifying the requirements for an economic and legal considerations framework for medical travel. These are based on a non-systematic, targeted literature review. Legal considerations include the standardizations of visa procedures, implementation of international quality standards, accreditation of health-care providers, and third-party agencies, and the protection of the local population, and the monitoring of information provided to patients to ensure informed decision making. Economic considerations include a dynamic approach to pricing that is fair to the local population. This list is not definitive, and we welcome further empirical research from economic and legal perspectives to utilize and build upon this framework.

## Well-Being

We were pleased to include a review highlighting the importance of seeing medical travel’s potential value beyond treatment. In the work by Záliš et al. a clear argument is presented for ensuring that the ultimate outcome of interest must be the wider impact on well-being, not simply volumes of patients receiving care. This is critical as it speaks directly to the trade-off policymakers must make between access and outcomes. Instances where faster or lower-cost care abroad creates risks to well-being that outweigh the gains are rightly outlined. We would hope this piece spurs debate and discussion regarding the topics it raises and their wider implications.

## Future Considerations

While access to care is clearly a positive incentive for travel, it is not without risks, even beyond the clinic. Well-being literature (5, 6) highlights the importance of social support in recovery and the idea of traveling abroad for care would seem to ignore this. Therefore, wider impacts on well-being should build on the evidence generated, as will those employer insurance plans that include medical travel.

The themes of this Research Topic are not exhaustive but rather serve to lay foundations for evidence required in future developments for medical travel policy. For research to be effective, evidence will have to be gathered with consideration of all the above elements, as well as potential gains or opportunities to improve access, quality of care, and health equity. There are also wider considerations that span beyond health services, including the development of pricing structures, engagement with third parties (e.g., visa services, travel industry), and stratified evidence based on treatments, regions, and clinicians, among many. Ultimately, it is clear that the challenge of medical travel is not only cross-national, but cross-sectoral: research should generate information for patients, clinicians, industry, and policymakers alike. Only then can the economic and medical benefits be responsibly explored through wider medical travel programs.

## Author Contributions

All authors listed have made substantial, direct, and intellectual contribution to the work and approved it for publication.

## Conflict of Interest Statement

The authors declare that the research was conducted in the absence of any commercial or financial relationships that could be construed as a potential conflict of interest.
